# Corrigendum: Towards automatic pulmonary nodule management in lung cancer screening with deep learning

**DOI:** 10.1038/srep46878

**Published:** 2017-09-07

**Authors:** F. Ciompi, K. Chung, S. J. van Riel, A. A. A. Setio, P. K. Gerke, C. Jacobs, E. T. Scholten, C. Schaefer-Prokop, M. M. W. Wille, A. Marchianò, U. Pastorino, M. Prokop, B. van Ginneken

Scientific Reports 7: Article number: 46479; 10.1038/srep46479 published online: 04 19 2017; updated: 09 07 2017.

This Article contains errors. The legend of Table 3 is incorrect.

‘**Nodule classification performance in terms of accuracy and F-measure per nodule type.** Results for each pair of human observer O*i* vs. O*j* and for observers versus the computer on the *test*_*OBS*_ dataset (167 nodules) are reported. Averages of measures across observers and across computer-observers are also indicated. The additional class “not a nodule” is added to observers since they could exclude nodules during the observer study. The performance of the system on the *test*_*ALL*_ set (639 nodules) is also reported. In this case, annotations from DLCST radiologists (O4) are considered as the reference standard’.

should read:

‘**Nodule classification performance in terms of accuracy and F-measure per nodule type.** Results for each pair of human observer O*i* vs. O*j* and for observers versus the computer on the *test*_*OBS*_ dataset (167 nodules) are reported. Averages of measures across observers and across computer-observers are also indicated. The additional class “not a nodule” is added to observers since they could exclude nodules during the observer study’.

Additionally, the title of Table 4 is incomplete.

‘Comparison of classification performance in terms of accuracy and F-measure when the considered methods are: (1) features based on pixel intensity of patches and linear SVM classifier, (2) features learned from raw nodule patches using the unsupervised learning approach proposed in ref. 34 and linear SVM classifier, (3) the proposed deep learning approach using ConvNets working at 1, 2 and 3 scales’.

should read:

‘Comparison of classification performance on the *test*_*ALL*_ set (639 nodules) in terms of accuracy and F-measure when the considered methods are: (1) features based on pixel intensity of patches and linear SVM classifier, (2) features learned from raw nodule patches using the unsupervised learning approach proposed in ref. 34 and linear SVM classifier, (3) the proposed deep learning approach using ConvNets working at 1, 2 and 3 scales. In these experiments, annotations from DLCST radiologists (O4) are considered as the reference standard’.

